# Evaluating Risk-Adjusted Hospital Performance Using Large-Scale Data on Mortality Rates of Patients in Intensive Care Units: A Flexible Semi-Nonparametric Modeling Approach

**DOI:** 10.1109/JTEHM.2023.3257179

**Published:** 2023-03-14

**Authors:** Yakun Liang, Xuejun Jiang, Bo Zhang

**Affiliations:** Department of Statistics and Data ScienceSouthern University of Science and Technology Shenzhen 518055 China; Department of Neurology and ICCTR Biostatistics and Research Design CenterBoston Children’s Hospital, Harvard Medical School Boston MA 02115 USA

**Keywords:** Hospital random effects, ICU mortality, repeated measures, risk adjustment, zero-inflated models

## Abstract

Background and objective: Significant variability in the quality of healthcare supplied by hospitals is drawing broad attention from the United States Centers for Medicare and Medicaid Services. The primary issue is to evaluate hospital performance based on patient outcomes. Generalized linear random-effects models are a promising analytical tool for evaluating hospital performance. However, hospital compare data often violate the classical assumptions of normality on random effects and linearity representation on transformed conditional mean structures in these models. Methods: In this article, we proposed and tested the performance of a class of hospital compare models that embraces nonparametric mean structures with semi-nonparametric hospital random effects. Such models were further improved and integrated into a zero-inflated model. $\mathtt {SAS}$ programs to implement these newly proposed hospital compare models were thoroughly developed. The $\mathtt {SAS}$ programs are freely available via a GitHub (https:\\www.GitHub.com) repository. Results: We demonstrate the robustness of the proposed hospital compare models by conducting intensive empirical studies. Flexible semi-nonparametric random effects and functional fixed-effects mean structure were used to analyze patient mortality in a large-scale intensive care unit data set. After applying the proposed models to assess standardized modality rates and address patient-mix variability across hospitals, we detected those underperforming hospitals with higher mortality rates. Conclusions: Our research findings highlight how constructing advanced assessment tools for hospital performance could support better decision-making at the administrative and public levels. The proposed hospital compare models are comprehensive in their capacity to identify patterns of hospital random effects and to convey the variability in healthcare quality with powerful accuracy and interpretability.

## I. Introduction

The Centers for Medicare and Medicaid Services (CMS) of the United States rely on various performance metrics, including mortality rates, readmission rates, and healthcare-associated infection rates, to provide the administration and general public the information regarding the quality of hospitals [1]. Measuring and improving hospital quality has quickly become a fundamental focus of clinicians and policymakers alike. Over the last decade, the CMS have implemented national value-based purchasing programs, which use Medicare provider payments to encourage and incentivize hospitals to deliver higher quality care with respect to clinical processes, patient experiences and outcomes, and treatment efficiency [2]. For instance, since heart failure, acute myocardial infarction, and pneumonia have all led to greater hospitalization and death rates in recent years, the CMS now ranks hospitals and imposes financial penalties according to their 30-day mortality rates for these admitted critical patients [3].

Inferior treatment can have substantial impacts on individual patients and consequently on hospital performance. In 2022, the U.S. News & World Report organization reported that due to age, physical ailments, or other chronic conditions, nearly two million hospital inpatients faced the prospect of surgery or special care, posing either unusual technical challenges that must be overcome or a significantly increased risk of harm or death [4]. Public reports of hospitals’ performance not only support their administrative management but also assist public health decision-making pertinent to nationwide health policy, with hospital rankings now a tool used by prospective patients to find sources of skilled medical treatment [5]. Hence, the reliable assessment and disclosure of healthcare providers’ clinical performance within hospitals has emerged as a crucial tactic for monitoring and improving the quality of the United States (U.S.) hospital care.

In hospitals, their intensive care units (ICUs) are unique units with concentrated resources to treat critically ill patients in dire need of radical, life-saving treatments. In the U.S., these ICUs together spend ${\$}81.7$ billion, and this total is trending upward since 2005 with accounting for 13.4% of hospital costs and 4.1% of national health expenditures [6]. Meanwhile, intensive care resources in the U.S. are limited [7]. Therefore, it is imperative to simultaneously improve both the care provided to patients in the ICUs and the cost-effectiveness of the critical care services. Uncovering the key linkages between critical care services and clinical outcomes can be very useful for optimizing the value of certain treatments and evaluating the performance of U.S. hospitals.

### A. Motivations

Mortality in the ICUs can be viewed as a proxy for a hospital’s impact on the well-being of its patients. Whether mortality occurs in the ICUs is significantly influenced by the care provided by the hospitals, as well as demographics, comorbidities, severity of illness, various vital signs, and laboratory test results. Several traditional severity assessment systems [8], including Acute Physiology and Chronic Health Evaluation (APACHE), Mortality Probability Model (MPM), and Simplified Acute Physiology Score (SAPS), are routinely used to assess mortality risk among patients in the ICUs. In these assessment tools, risk adjustment has not been performed to adjust for various confounding factors associated with individuals and hospitals. It has been shown that building accurate risk-adjusted hospital compare models based on ICU mortality rates is of paramount importance. Doing so can enable us to better understand which factors contribute to the diversity of health outcomes, in addition to revealing potential superior and inferior performing hospitals.

In assessing hospital performance and ranking the hospitals, previous researchers have overwhelmingly relied upon the generalized linear mixed models (GLMMs) [9], [10], [11] due to the interpretability of their model structure, in which random effects are used to account for unobserved heterogeneity across the grouped individuals with a hospital. Usually, these hospital-specific random effects are assumed to be normally distributed in the GLMMs as before. However, this may lead to a misspecification of the distribution of the random effects, if the underlying truth is not a normal distribution, and thereby further induce biases in estimation and prediction. To reduce such biases, a more flexible nonparametric distribution for characterizing random effects is evidently required and desired. In this article, we explore a framework for risk-adjusted hospital performance by proposing a flexible semi-nonparametric (SNP) modeling approach to the hospital random effects in the GLMMs as hospital compare models, and meanwhile integrate the B-spline nonparametric functional representation into the conditional mean structure of the GLMMs. Such GLMMs for hospital compare and assessment are further improved by a zero-inflated modelling approach to characterize excessive zero outcomes.

### B. Contributions

The major contributions of this research are briefly summarized as follows:

1) A new framework for logistic random-effects models is proposed to measure hospital performance in terms of ICU mortality. The classical GLMMs are extended to have hospital fixed effects represented by an additive term of B-spline basis functional structure and to have the semi-nonparametric random effects with an SNP density. The goal of the extension is to more accurately capture the heterogeneity across hospitals and a risk factor that is a nonlinear addition term in the mean structure.
2) The zero-inflated structure is introduced for modeling mortality-risk-free individuals in the ICU data. To our best knowledge, it is the first hospital compare modeling framework that explicitly caters to random effects nonnormality, conditional mean nonlinearity, and response zero-inflation in large-scale hospital compare data of patient outcomes.
3) The utility of the newly proposed models and the robustness of their model fitting are demonstrated by extensive simulation studies. We analyzed a large-scale ICU data set and evaluated the risk-adjusted hospital performance by direct and indirect standardization of mortality rates. The corresponding $\mathtt {SAS}$ programs for implementing the proposed hospital compare models are provided on the GitHub platform (available at https://github.com/YakunLab/SNP-MM).

The rest of this article is organized as follows. In Section II, we summarize recent research on the analytical models used in the field of hospital compare and ranking. In Section III, we propose the SNP random-effects models with a nonparametric additive term and a zero-inflated structure. In Section IV, we introduce direct and indirect standardization of mortality rates. In Section V, we conduct simulation studies, to compare the performance of the proposed models vis-à-vis existing competitors. In Section VI, we illustrate the utility of the proposed models through an analysis of a large-scale empirical ICU mortality data set from the U.S. Section VII concludes this article with a brief discussion of its findings.

## II. Related Work

### A. Current Practice

In hospital compare studies, the patients can be clustered by hospital. These observations are denoted by $(Y_{hj}, \boldsymbol {X}_{hj}, \boldsymbol {V}_{h})$ for $j=1, 2, {\dots }, n_{h}$ and $h=1, 2, {\dots }, H$, where $Y_{hj}$ is the response variable for patient $j$ in hospital $h$; $\boldsymbol {X}_{hj}$ is the covariate vector representing the patient’s attributes; and $\boldsymbol {V}_{h}$ is the vector of hospital-specific attributes, such as the number of admitted patients to the ICU. Here, we focus on modeling the binary event $Y_{hj}$ for each patient, assigning a value of 1 or 0 to indicate whether or not event (i.e., mortality) occurred.

The assessment model used to estimate the hospital mortality rates among patients tries to infer patient and hospital effects [9]. It fits a random-effects logistic regression:

\begin{equation*} \text {logit}(p_{hj}) = a + b_{h} + \boldsymbol {X}^{\top }_{hj} \boldsymbol {\beta }, \tag{1}\end{equation*}

where the logit link is $\log \left ({p_{hj}/(1-p_{hj})}\right)$, and $p_{hj} = P(Y_{hj} = 1| \boldsymbol {X}_{hj}, b_{h})$ is the mortality rate for the $j$-th patient within the $h$-th hospital, $j=1, {\dots }, n_{h}$, $h=1, {\dots }, H$. As such, the patient effect is determined by the common fixed-effect vector $\boldsymbol {\beta }$. When subsuming the fixed intercept $a$ into the hospital random effect, $b_{h}$, it is typically assumed that $b_{h}$ follows a normal distribution $N(a, \sigma ^{2})$ and reflects all basic risks posed by hospitals. However, because this model ignores the plausible impact of hospital-associated factors, the Gaussian random intercept makes it difficult to interpret the treatment effects of hospitals. Taking a Bayesian perspective, Silber et al. [10] improved the above model (1) to allow the $b_{h}$ to relate to hospital attributes $\boldsymbol {V}_{h}$, by assuming the expectation of $b_{h}$ given $\boldsymbol {V}_{h}$ to be a function of $\boldsymbol {V}_{h}$; i.e., $b_{h}| \boldsymbol {V}_{h}\sim N(a(\boldsymbol {V}_{h}), \sigma ^{2})$. In practice though, the $\boldsymbol {V}_{h}$ of interest usually is the logarithm of the number of admitted patients, or the number of beds, for the hospital. In a similar way, to account for any potential relationship between mortality and hospital attributes, George et al. [11] modified the hospital compare model as follows:

\begin{equation*} \text {logit}(p_{hj}) = b_{h} + \boldsymbol {V}^{\top }_{h} \boldsymbol {\alpha } + \boldsymbol {X}^{\top }_{hj} \boldsymbol {\beta },\end{equation*}

whereby the hospital effect is now specified by the fixed-effect vector $\boldsymbol {\alpha }$, the distribution of $b_{h}$ depends on hospital attributes $\boldsymbol {V}_{h}$, equivalent to $b_{h}| \boldsymbol {V}_{h}\sim N(a(\boldsymbol {V}_{h}),\sigma ^{2}(\boldsymbol {V}_{h}))$. Bayesian methods, however, require that careful attention paid to the prior distribution as well as a follow-up sensitivity analysis.

Some work has suggested ways to improve the hospital compare model of George et al. [11]. For example, Caroff et al. [12] showed that the ranking model of hospitals provides a better risk adjustment with higher accuracy when claims-based comorbidities and patient-level electronic health records are both considered. Further, the work by George et al. [11] also illustrated some prospective approaches to improve the modeling of mortality rates. This includes introducing robust parametric families for random-effect distributions to mitigate the influence of extreme values, or adopting a nonparametric prior for the random effect to obtain more accurate fits in a wide variety of realistic situations. In general, besides adding more explanatory variables or deciding on one or more random effects is also a key step in the GLMMs modeling.

### B. Hospital Random Effects

Hospital compare or hospital ranking data which are naturally clustered are commonly analyzed and interpreted using mixed-effects models, in which the random effect follows a Gaussian distribution, but this routine assumption is restrictive. Although some estimators are robust to misspecification of the random-effects distribution, there is inconsistency in the maximum likelihood estimators when violating the distributional assumptions of normality [13], [14]. Classical random-effects models can suffice in some fields, but they fall short for complex scenarios.

Several researchers have proposed methods to relax this normality assumption by relaying instead on a semi-parametric projection method [15] or applying an alternative scale mixture of skew-normal distributions [16]. These attempts to model random effects with parametric families do show some promising efficiency gains in fitting a more flexible model when their normality assumption is violated. By representing the smooth density of random effects in a Hermite expansion [17], the SNP method is capable of estimating the random effect of a GLMM with computational efficiency, which captures heterogeneity flexibly under a variety of extensions [18], [19]. In this article, we adopt the SNP structure for designating random effects to identify latent hospital effects.

### C. Risk Adjustment

The impetus for adjusting risk is to quantify the contribution of relevant variables to mortality while controlling for any potential risk factors [20]. To build a hospital compare model that can rank hospitals fairly, a correct risk adjustment is essential, especially when the underlying treatments of hospitals differ in how they significantly influence a disease. Risk adjustment can be done in two main ways, via standardized indirect or direct methods [11]. Notably, hospital volume (or the number of admissions) plays an instrument role in quality assessments of hospitals. Being easily detectable from ICU data, a low hospital volume may indicate an inadequate risk adjustment, which could put small hospitals at a disadvantage [20]. Thus, both the patient profile and hospital volume are crucial factors in the risk adjustment applied to mortality data.

### D. Drawbacks

Despite the above advances, current hospital compare models still harbor several notable limitations:

1) Do the normality assumption on random effects and the conditional linear mean structure suffice? The latent random effects may not follow a normal distribution. Thus, a normality assumption on their latent distribution can cause problems in inference and prediction in hospital compare random-effects models. In addition, nonlinear effects of risk factors may be neglected in these models. For example, Mullah et al. [21] precisely estimated the perinatal mortality in twins when considering nonlinear associations between their birth weight and gestational age response. Identifying nonlinear effects on mortality is beneficial for accurate inference and prediction.
2) How to address the issue of excessive zeros in mortality outcomes? The prevalence of rare positive or mortality cases (i.e., nonzero cases in responses) naturally exists in hospital compare studies. Caroff et al. [12] suggested that low-volume treatment procedures and sporadic infections at the colon surgical site are barriers to effectively comparing the performance of hospitals. Dealing with the issue of excessive zeros raised from rare nonzero cases can no longer be avoided and is necessary for alleviating potentially hidden biases in the inference and prediction provided by the GLMMs.

There seems to be ample room for further research to tackle these challenges head-on. This article is devoted to providing the first extensive study in the hospital compare research aiming to explore analytical comparison models for hospitals.

## III. Enhanced Hospital Compare Random- Effects Models

The proposed hospital compare models described here are divided into two subsections. Beginning with the model (1), in Section III-A, we propose hospital compare GLMMs that combine the SNP hospital random effect and a nonparametric hospital fixed effect, to better distinguish hospital effects. In Section III-B, we propose the zero-inflated Bernoulli (ZIB) hospital compare models by applying the zero-inflation approach to characterize excessive zeros in mortality outcomes in hospital compare data that contain a large proportion of zeros that are raised by the group of patients with zero probability of death during ICU care.

### A. The Hospital Compare Models With an SNP Random Effect

#### 1) From Linear to Nonlinear Transformed Conditional Mean Structures

The random effects in the GLMMs have the role of identifying the remaining unexplained hospital-related heterogeneity in the outcomes. However, the transformed conditional mean structure may be beyond linear representation as presented by George et al. [11]. Because nonparametric term in the mean structure is capable of inferring the appropriate complexity between the outcomes and the predictors in the GLMMs, a more flexible model for mortality status $Y_{hj}$ was created as follows [11]:

\begin{equation*} \text {logit}(p_{hj}) = b_{h} + f(V_{h})+ \boldsymbol {X}^{\top }_{hj} \boldsymbol {\beta }, \tag{2}\end{equation*}

where the logit link is $\log \left ({p_{hj}/(1-p_{hj})}\right)$, and $p_{hj} = P(Y_{hj} = 1| \boldsymbol {X}_{hj}, V_{h}, b_{h})$ is the probability of mortality of the $j$-th patient within the $h$-th hospital, $j=1, {\dots }, n_{h}$, $h=1, {\dots }, H$. Here, $f(V_{h})$ denotes a flexible nonparametric fixed effect with respect to a hospital attribute $V_{h}$; $\boldsymbol {\beta }$ is the patient fixed effect related to covariates $\boldsymbol {X}_{hj}$; and $b_{h}$ is the random hospital effect whose distribution is unknown. We do not assume any specific functional form of $f(V_{h})$ other than smoothness. For identifiability, it is stipulated that $E(f(V_{h})) = 0$.

To construct a flexible nonparametric fixed effect $f(V_{h})$, we take the B-spline approximation [22], a common computationally efficient technique. With compact support, each given basis function in the B-spline approximation is nonzero over a span of a small number of distinct knots. Without a loss of generality, we assume that $V_{h}\in [{0,1}]$. Let $\tilde { \boldsymbol {B}}(V_{h}) = (\tilde {B}_{1}(V_{h}), {\dots }, \tilde {B}_{m_{n}}(V_{h}))^{\top }$ be a set of B-spline basis functions, where $m_{n}=q+d+1$, with $d$ being the degree of the polynomial and $q$ being the number of quasi-uniform interior knots. Further details on the construction of B-spline basis functions can be found in [22].

Next, let $\boldsymbol {B}(V_{h}) = (B_{1}(V_{h}), {\dots }, B_{m_{n}}(V_{h}))^{\top }$, where $B_{l}(V_{h})=\tilde {B}_{l}(V_{h}) - H^{-1}\sum _{\mathcal {H}=1}^{H} \tilde {B}_{l}(V_{\mathcal {H}})$. Such a transformation to the basis functions is required to render the model identifiable and to restrict the subspace of splines in the $(q+d)$-dimension. For $f(V_{h})$, there exists the unique vector $\boldsymbol {\gamma }=(\gamma _{1}, \ldots, \gamma _{m_{n}})^{\top }$ which satisfies the following:

\begin{equation*} f(V_{h}) \approx \boldsymbol {B}(V_{h})^{\top } \boldsymbol {\gamma }. \tag{3}\end{equation*}

Concerning the expansion of each sample $\boldsymbol {B}(V_{h})$ as a part of the design matrix, $\boldsymbol {\gamma }$ can be estimated by following the estimation procedures in the classical GLMMs because

\begin{equation*} \text {logit}(p_{hj}) \approx b_{h} + \boldsymbol {B}(V_{h})^{\top } \boldsymbol {\gamma } + \boldsymbol {X}^{\top }_{hj} \boldsymbol {\beta }. \tag{4}\end{equation*}

#### 2) From Normal to SNP Random Effects

The hospital effects of the model (2) is composed of the fixed functional term $f(V_{h})$ and the random hospital effect $b_{h}$. We assume that $b_{h}$ is independent across the $h$, for $h=1, {\dots },H$, and that it can be expressed as the SNP representation of

\begin{equation*} b_{h} = \sigma z_{h} + a, \tag{5}\end{equation*}

where $a$ is a location constant, $\sigma $ is a scale constant, and $z_{h}$ is a random variable. If $z_{h}$ follows the standard Gaussian density $N(0, 1)$, then $b_{h}\sim N(a,\sigma ^{2})$, which exactly satisfies the classical normality assumption in model (1). To minimize the induced biases associated the misspecified random-effect density, the probability density of random effects satisfying certain smoothness restrictions and differentiability conditions can be represented by an infinite Hermite series expansion plus a lower bound on its tail behavior [17]. Here, we build upon the idea of truncated Hermite expansion [18] to characterize the random effect $b_{h}$ in the proposed hospital compare model (2).

Let the density of $z_{h}$ be $h_{K}(z_{h})$. We assume $h_{K}$ can be represented by a standard SNP density,

\begin{align*} {h_{K}(z_{h})} \!\propto \! P^{2}_{K}(z_{h})\phi (z_{h}) = \left \{{\sum _{j=0}^{K}\xi _{j}z_{h}^{j}}\right \}^{2}\phi (z_{h}) \!=\! \left ({\boldsymbol {z}_{h}^{\top } \boldsymbol {\xi } }\right)^{2}\phi (z_{h}),\!\! \tag{6}\end{align*}

where the fixed integer $K$ is the degree of truncation, this expressing the order of the polynomial $P_{K}$, $\boldsymbol {z}_{h}=(z_{h}^{0},z_{h}^{1}, {\dots },z_{h}^{K})^{\top }$ is a vector of the random variable $z_{h}$; $\boldsymbol {\xi }=(\xi _{0}, {\dots },\xi _{K})^{\top }$ is the vector of coefficients; and $\phi (\cdot)$ is the density function of the standard Gaussian distribution. Accordingly, $K$ is a tuning parameter that governs the degree of flexibility for density $h_{K}$. For $h_{K}(z_{h})$ to be a density that imposes the condition of $E(P^{2}_{K}(z_{h}))=1$, the polynomial coefficients $\boldsymbol {\xi }$ can be estimated by reparameterization in a polar coordinate transformation method; for that, see [18] for details.

As the density of $h_{K}$ is represented by equation (6), the SNP density of $b_{h}$ can be derived as follows:

\begin{equation*} g_{K}(b_{h}; \boldsymbol {\xi }, a, \sigma)=P_{K}^{2}(z_{h}; \boldsymbol {\xi }) N(b_{h}; a, \sigma ^{2}) \tag{7}\end{equation*}

where $z_{h}=\sigma ^{-1}(b_{h}-a), N(b_{h}; a, \sigma ^{2})$ is the Gaussian density with mean $a$ and variance $\sigma ^{2}$. Therefore, the SNP density does not require that $E(b_{h})=0$. When $K=0$, $P_{K}$ only has a constant coefficient of $\xi _{0}=1$. Then SNP density for random effect reduces to a Gaussian that follows the normality assumption in GLMMs. A larger degree $K$ value allows for more flexibility in detecting heterogeneity, but it severely limits the efficiency. Thus, choosing a small $K$ value can usually lead to a reasonable trade-off between computational complexity and goodness of fit of the model. In practice, it is advised to use information criteria such as AIC and BIC to find an optimal $K$.

### B. The ZIB Hospital Compare Models

#### 1) From One-Part Models to Zero-Inflated Models

For hospital compare data with excessive zeros that cause class-imbalance, oversampling is a popular technique but can overgeneralize minority instances due to indistinguishable overlapping [23]. However, this approach is not beneficial for risk assessment. To avoid possible biases in inference and prediction, Hall [24] introduced zero-inflated regression for count data having an excessive number of zeros. Therefore, when too many zeros are observed in responses, we recommend identifying risk-free patients (i.e., the patients with zero probability of death during ICU care) by the ZIB structure to improve the proposed hospital compare model (2). The original ZIB model assumes the mortality status $Y_{hj}$ results from the following process,

\begin{align*} Y_{hj} \sim \begin{cases} \displaystyle 0, & { \text {with probability }} 1-\rho _{hj}, \\ \displaystyle \mathrm {Bernoulli}(\pi _{hj}), & { \text {with probability }} \rho _{hj}, \end{cases} \tag{8}\end{align*}

where $1-\rho _{hj}$ models the excessive zeros and describes the proportion of risk-free patients, while $\pi _{hj}$ models the probability of mortality for patients who are still at risk.

To explain the probability $\rho _{hj}$, we first introduce another binary variable: $S_{hj}$. It denotes whether the individual is either still at risk of mortality ( $S_{hj} = 1$) or at no risk of mortality ( $S_{hj} = 0$). Nevertheless, zero responses (i.e., $Y_{hj} = 0$) will include both fixed zeros from the risk-free group in addition to random zeros from the at-risk group, whose members are unknown. To distinguish those patients having no risk of mortality, we propose the following:

\begin{equation*} \text {logit}(\rho _{hj}) = \boldsymbol {T}^{\top }_{hj} \boldsymbol {\theta }, \tag{9}\end{equation*}

where $\rho _{hj} = P(S_{hj} = 1| \boldsymbol {T}_{hj})$, $\boldsymbol {T}_{hj}$ is a covariate vector constructed by the risk-related factors of a given patient, and $\boldsymbol {\theta }$ is a vector of the corresponding unknown parameters. In practice, $\boldsymbol {T}_{hj}$ may overlap one another or even be identical to $\boldsymbol {X}_{hj}$. For a patient at risk of mortality (i.e., $S_{hj}=1$), we characterize the mortality-related factors by extending the model (2) in the way of

\begin{equation*} \text {logit}(\pi _{hj}) = b_{h} + f(V_{h}) + \boldsymbol {X}^{\top }_{hj} \boldsymbol {\beta }, \tag{10}\end{equation*}

in which $\pi _{hj} = P(Y_{hj} = 1| \boldsymbol {X}_{hj}, V_{h}, b_{h}, S_{hj})$. Further, the individual mortality rate is given by

\begin{align*} p_{hj}(\boldsymbol {X}_{hj}, \boldsymbol {T}_{hj}) = \,\, & P(Y_{hj} = 1| \boldsymbol {X}_{hj}, V_{h}, b_{h}, \boldsymbol {T}_{hj}) \\ = \,\, & P(Y_{hj} = 1| \boldsymbol {X}_{hj}, V_{h}, b_{h}, S_{hj})P(S_{hj} = 1| \boldsymbol {T}_{hj}) \\ = \,\, & \pi _{hj} \rho _{hj}. \tag{11}\end{align*}

#### 2) Likelihood Estimation

Suppose $(Y_{hj}, \boldsymbol {X}_{hj}, \boldsymbol {T}_{hj}, V_{h})$ is a sample of independent random vectors for the $j$-th patient in the $h$-th hospital, where $j=1,\ldots,n_{h}$, $h=1,\ldots,H$.Define $\boldsymbol {Y}=(\boldsymbol {Y}^{\top }_{1},\cdots, \boldsymbol {Y}^{\top }_{H})^{\top }$ with $\boldsymbol {Y}_{h}=(Y_{h1}, \cdots, Y_{hn_{h}})^{\top }$, then the likelihood function of $\boldsymbol {Y}$ is given by

\begin{align*} &\hspace {-1.3pc}\mathcal {L}(\boldsymbol {\beta }, \boldsymbol {\theta }, \boldsymbol {\gamma }; \boldsymbol {Y}) \\ = \,\, &\prod _{h=1}^{H} \int \prod _{j=1}^{n_{h}} f_{Y_{hj}| b_{h}}(Y_{hj}| b_{h}; \boldsymbol {\beta }, \boldsymbol {\theta }, \boldsymbol {X}_{hj}, \boldsymbol {T}_{hj}, V_{h}) g_{b_{h}}(b_{h}) \text {d}b_{h} \\ = \,\, & \prod _{h=1}^{H} \int \exp \Biggl \{ \sum _{j=1}^{n_{h}} \Big [Y_{hj} \left ({\tau _{hj} +\kappa _{hj} }\right) \\ &+\, (1-Y_{hj})\log \left ({1+e^{\tau _{hj}}+e^{\kappa _{hj}}}\right) \\ &-\, \log \left ({1+e^{\tau _{hj}}}\right) - \log \left ({1+e^{\kappa _{hj}}}\right) \Big] \Biggl \} g_{b_{h}}(b_{h}) \text {d}b_{h}, \tag{12}\end{align*}

where $\tau _{hj} = \boldsymbol {T}^{\top }_{hj} \boldsymbol {\theta }$, $\kappa _{hj} = b_{h} + \boldsymbol {B}(V_{h})^{\top } \boldsymbol {\gamma } + \boldsymbol {X}^{\top }_{hj} \boldsymbol {\beta }$ with $\boldsymbol {B}(V_{h})^{\top } \boldsymbol {\gamma }$ being the B-spline basis expansion of $f(V_{h})$ in model (10), and $g_{b_{h}}(b_{h})$ is the density of $b_{h}$ as defined in (7). In the $\mathtt {SAS}$ programs, adaptive Gaussian quadrature can be employed to numerically evaluate $\mathcal {L}(\boldsymbol {\beta }, \boldsymbol {\theta }, \boldsymbol {\gamma }; \boldsymbol {Y})$. Maximum likelihood or restricted maximum likelihood estimates of unknown parameters can be obtained when combined with a nonlinear optimization algorithm, such as the quasi-Newton or Newton-Raphson.

## IV. Standardized Mortality Rates for Hospital Compare Reporting

Indirect and direct standardization of mortality rates is a central concept in hospital compare and ranking. Support the mortality events $Y_{hj}$, $j=1, {\dots },n_{h}$, $h=1, {\dots },H$, and then the overall average event rate can be obtained by $OR = N^{-1}\Sigma _{h,j} Y_{hj}$, where $N = \Sigma _{h} n_{h}$, and the observed mortality rate for hospital $h$ is $OR_{h} = n_{h}^{-1}\Sigma _{j} Y_{hj}$. Due to differences in the number of patients served in hospitals and attributes of patient populations across hospitals, comparability is lacking when estimating hospital-specific mortality rate by simply averaging the estimated patient rates from the proposed models (2) and (11).

Since the mortality rate is modeled by related risk factors, a more accurate evaluation of mortality rates should eliminate patient case-mix effects via some type of standardization [20]. To achieve this goal, we first introduce the evaluation method for the probability of death for one patient seeking treatment at a specific hospital. Based on model (2), if a patient with covariates $\boldsymbol {\mathcal {X}}$ received treatment at hospital $\mathcal {H}$, the mortality rate prediction is given by

\begin{equation*} p_{\mathcal {H}}(\boldsymbol {\mathcal {X}}) = \text {logit}^{-1} \left ({b_{\mathcal {H}} + f(V_{\mathcal {H}}) + \boldsymbol {\mathcal {X}}^{\top } \boldsymbol {\beta }}\right), \tag{13}\end{equation*}

where $\boldsymbol {\beta }$ is the usual patient effect associated with $\boldsymbol {\mathcal {X}}$, $f(V_{\mathcal {H}})$ is the hospital fixed effect, $b_{\mathcal {H}}$ is the random effect for hospital $\mathcal {H}$, and $\mathcal {H} = 1, \ldots,H$. When $\boldsymbol {\mathcal {X}}= \boldsymbol {X}_{hj}$ and $\mathcal {H} = h$, formula (13) determines the mortality rate of the $hj$-th patient in the observations who received treatment at hospital $h$. Indirect and direct standardization of mortality rates based on (13) are introduced below.

### A. Indirectly Standardized Mortality Rates

The mortality rate for a specific hospital $h$ is assessed contingent on patient cases formerly received and served, which is a function of observations with patient attributes $\boldsymbol {X}_{h1}, {\dots }, \boldsymbol {X}_{hn_{h}}$. An indirectly risk-standardized, hospital-specific mortality rate is presented in the CMS White Paper [1] is given by

\begin{equation*} P^{IS}_{h} = (P_{h} / E_{h}) \times OR, \tag{14}\end{equation*}

where $P_{h} = n_{h}^{-1} \sum _{j=1}^{n_{h}} p_{h}(\boldsymbol {X}_{hj})$ with $\mathcal {H} = h$ as given in (13), which is an average of the mortality rate prediction $p_{h}$ and measures the mortality risk of hospital $h$; $E_{h}$ is an average of the expected mortality rate when presupposing the same type of patients were treated in the target hospitals at the “national-level”. Since $E_{h}$ conveys the national-level performance of care, a more suitable option is that proposed by George et al. [11], which expands the scope of “national-level” hospitals to include all hospitals within the current hospital compare data as follows:

\begin{equation*} E_{h} = \frac {1}{n_{h}} \sum _{j=1}^{n_{h}} \left [{ \frac {1}{H} \sum _{\mathcal {H} = 1}^{H} p_{\mathcal {H}}(\boldsymbol {X}_{hj})}\right]. \tag{15}\end{equation*}

Thus, indirect standardization of mortality rates relay on the perspective of received patients and investigate differences in their outcomes between past and “national-level” services.

As in [11], low-volume hospitals serving relatively fewer patients have a higher-than-typical risk, with their proportion $P_{h} / E_{h}$ being significantly larger than one. The denominator of (14), $E_{h}$, takes on a benchmarking role and conveys the systemic risk faced by patients served in hospital $h$. Yet, when evaluating the mortality risk of individuals in other hospitals, equation (15) assigns the same weight to each patient under consideration. Because high-volume hospitals are more popular among prospective patients, the $E_{h}$ given by (15) should be adjusted further, to balance the subjective choices of patients. To that end, we propose a weighted risk in hospital $h$ given by

\begin{equation*} E_{h} = \frac {1}{n_{h}} \sum _{j=1}^{n_{h}} \left [{\sum _{\mathcal {H} = 1}^{H}w_{\mathcal {H}} p_{\mathcal {H}}(\boldsymbol {X}_{hj})}\right], \tag{16}\end{equation*}

where $\mathcal {H} = 1, \ldots,H$ and $\sum _{{\mathcal {H}}=1}^{H}w_{\mathcal {H}}=1$. In practice, these weights can be specified through the hospital’s volume.

### B. Directly Standardized Mortality Rates

The mortality rate for a specific hospital $h$ is assessed contingent on patient cases might get served there, which is a function of all patients within the investigation with attributes $\boldsymbol {X}_{\mathcal {H}j}$, $j=1, {\dots },n_{\mathcal {H}}$, and $\mathcal {H}=1, {\dots },H$. Denoted as $P^{DS}_{h}$, the directly risk-standardized, hospital-specific mortality rate [11] is given by

\begin{equation*} P^{DS}_{h} = \frac {1}{N} \sum _{\mathcal {H} = 1}^{H} \sum _{j=1}^{n_{h}} p_{h}(\boldsymbol {X}_{\mathcal {H}j}), \tag{17}\end{equation*}

which does not rely on $E_{h}$. Here, the direct standardization of mortality rates adopts the perspective of evaluated hospitals and directly eliminate patient-mix effects by averaging the mortality rates of all patients had they been treated at hospital $h$.

## V. Simulation Experiments

In this section, we carry out extensive simulation studies to illustrate the performance of the proposed hospital compare models. In Section V-A, we demonstrate by conducting simulation experiments that the classical normality assumption for the random effects and the linearity assumption for the fixed effects struggle to cope with complex real-world scenarios, and further validate the flexibility and robustness of the SNP random effect and the functional fixed effect in the proposed hospital compare model (2). In Section V-B, we investigate the utility of the proposed ZIB hospital compare models (9) –(10) for analyzing data with excessive zeros. All these experiments are conducted in $\mathtt {SAS}$ version 9.4.

### A. Utility of Flexible Hospital Random and Fixed Effects

To reveal the importance of introducing semi-parametric and semi-nonparametric modeling approaches into hospital compare GLMMs, we focus and report the results from statistical simulations conducted with the following hospital compare model:

\begin{align*} \log \left ({\frac {p_{hj}}{1-p_{hj}}}\right) = b_{h} + f(V_{h}) + {X}^{\top }_{hj1}\beta _{1}+ {X}^{\top }_{hj2}\beta _{2}+ {X}^{\top }_{hj3}\beta _{3},\tag{18}\end{align*}

in which the vector of covariates, $\boldsymbol {X}_{hj}^{\top }=(X_{hj1},X_{hj2},X_{hj3})$, comes from $N(0, \boldsymbol {I}_{3})$, where $\boldsymbol {I}_{p}$ is a $p$ identity matrix. Parameters of patient effects are set as $\boldsymbol {\beta }=(\beta _{1},\beta _{2},\beta _{3})^{\top }=(3, 0.5, -5)^{\top }$. In the two scenarios described below, we respectively control the random effect $b_{h}$ and the functional fixed effect $f(V_{h})$, to illustrate the role of either component in the modeling. Values for the outcome $Y_{hj}$ after a medical event are obtained from $\mathrm {Bernoulli}(p_{hj})$ distribution, with $p_{hj}$ generated from (18).

A Monte Carlo simulation with 200 replications is conducted for the empirical analysis. Each data set consists of 500 hospitals. The number of patients $n_{h}$ for each hospital is the same, at 50. To measure the accuracy of function approximation, the integrated squared error (ISE) is used:

\begin{equation*} \mathrm {ISE}:=\frac {1}{H} \sum _{h=1}^{H}\left ({\hat {f}\left ({V_{h}}\right)-f\left ({V_{h}}\right)}\right)^{2}. \end{equation*}

Meanwhile, the relative distance (RD) is used to quantify the accuracy of the $\boldsymbol {\beta }$ parameter estimates, which is defined as

\begin{equation*} \mathrm {RD}:= \frac {\Vert \hat { \boldsymbol {\beta }}- \boldsymbol {\beta } \Vert }{\Vert \boldsymbol {\beta } \Vert }. \end{equation*}

The estimates of $E(b_{h})$ and $Var(b_{h})$ are then used to evaluate the performance of random-effect estimation.

#### 1) Random Effects

In this scenario, we generate the functional fixed effect as $f(V_{h}) = e^{-2V_{h}}\sin (6V_{h}) - 0.5748$, where $V_{h}\sim \mathrm {Uniform}(-1,1)$, $h=1, {\dots },H$. As in [13], the true density of $b_{h}$ covers a wide range of shapes, including symmetric, asymmetric, skewed, unimodal, and bimodal distributions. Seven types of distribution, each transformed to satisfy the mean zero and unit variance, are considered:

1) Normal: $N(0, 1)$
2) Uniform: $\mathrm {Uniform}(-\sqrt {3},\sqrt {3})$
3) Exponential: $\mathrm {Exp}(1) - 1$
4) Log-normal: $\mathrm {LogN}(0, 0.6937)-1.2720$
5) Symmetric mixture: $0.5N(-0.96, 0.28^{2})+0.5N\,\,(0.96, 0.28^{2})$
6) Asymmetric mixture: $\frac {2}{3}N\left({-\frac {2}{3}, \left({\frac {1}{3}}\right)^{2}}\right)+\frac {1}{3}N\left({\frac {4}{3}, \left({\frac {1}{3}}\right)^{2}}\right)$
7) Discrete: $P(b_{h}=-1) = P(b_{h}=1) = \frac {1}{2}$

We first fit the data using hospital compare model (2) with an SNP random effect, as defined in (7). This allows us to evaluate the flexibility of SNP estimation under different settings. To estimate the nonparametric $f(V_{h})$, we adopt cubic splines ( $d=3$) with three equally spaced interior knots ( $q=3$) for the B-spline approximation. Here, the $\mathtt {SAS}$ macro $\mathtt {SNP\_{}NLMM}$ is used to implement the model fitting; more information about this macro can be found in [25]. The BIC criterion is employed to determine the SNP structure of $b_{h}$. To investigate the impact of $b_{h}$’s misspecification on modeling, by way of comparison, we still use model (2) to fit the data but instead assume that $b_{h}$ follows the classical normal distribution $N(a,\sigma ^{2})$. All these results are reported in Table 1. In Table 1, to the left are results for the SNP method, while those under the classical normality assumption are on the right.

**TABLE 1:** Results of Different Random-Effect Distributions Under Mean Zero and Identical Variance. Standard Deviations are Shown in Parentheses

Distribution	SNP				Normal			
	$E(b_{h})$	$Var(b_{h})$	ISE	RD ( $\times 10^{-2}$)	$E(b_{h})$	$Var(b_{h})$	ISE	RD ( $\times 10^{-2}$)
Truth	0	1	0	0	0	1	0	0
$\begin{aligned} \begin{array}{c}\text{Normal}\\{}\end{array} \end{aligned}$	$\begin{aligned} \begin{array}{c}-0.0024 \\ \text{(0.09)}\end{array} \end{aligned}$	$\begin{aligned} \begin{array}{c}{0.9795}\\ \text{(0.08)}\end{array} \end{aligned}$	$\begin{aligned} \begin{array}{c}{0.0276}\\ \text{(0.013)}\end{array} \end{aligned}$	$\begin{aligned} \begin{array}{c}{0.0304}\\ \text{(0.036)}\end{array} \end{aligned}$	$\begin{aligned} \begin{array}{c} -0.0024 \\ \text{(0.09)}\end{array} \end{aligned}$	$\begin{aligned} \begin{array}{c} {0.9812}\\ \text{(0.08)}\end{array} \end{aligned}$	$\begin{aligned} \begin{array}{c} {0.0269}\\ \text{(0.013)}\end{array} \end{aligned}$	$\begin{aligned} \begin{array}{c} {0.0300}\\ \text{(0.036)}\end{array} \end{aligned}$
$\begin{aligned} \begin{array}{c}\text{Uniform}\\{}\end{array} \end{aligned}$	$\begin{aligned} \begin{array}{c}{0.0000}\\ \text{(0.09)}\end{array} \end{aligned}$	$\begin{aligned} \begin{array}{c}{0.9541}\\ \text{(0.11)}\end{array} \end{aligned}$	$\begin{aligned} \begin{array}{c}{0.0271}\\ \text{(0.012)}\end{array} \end{aligned}$	$\begin{aligned} \begin{array}{c}{0.0410}\\ \text{(0.058)}\end{array} \end{aligned}$	$\begin{aligned} \begin{array}{c} -0.0003 \\ \text{(0.09)}\end{array} \end{aligned}$	$\begin{aligned} \begin{array}{c} {0.9864}\\ \text{(0.07)}\end{array} \end{aligned}$	$\begin{aligned} \begin{array}{c} {0.0277}\\ \text{(0.013)}\end{array} \end{aligned}$	$\begin{aligned} \begin{array}{c} {0.0296}\\ \text{(0.034)}\end{array} \end{aligned}$
$\begin{aligned} \begin{array}{c}\text{Exponential}\\{}\end{array} \end{aligned}$	$\begin{aligned} \begin{array}{c}-0.0009 \\ \text{(0.09)}\end{array} \end{aligned}$	$\begin{aligned} \begin{array}{c}{0.9725}\\ \text{(0.14)}\end{array} \end{aligned}$	$\begin{aligned} \begin{array}{c}{0.0232}\\ \text{(0.012)}\end{array} \end{aligned}$	$\begin{aligned} \begin{array}{c}{0.0270}\\ \text{(0.033)}\end{array} \end{aligned}$	$\begin{aligned} \begin{array}{c} -0.0043 \\ \text{(0.09)}\end{array} \end{aligned}$	$\begin{aligned} \begin{array}{c} {0.9519}\\ \text{(0.14)}\end{array} \end{aligned}$	$\begin{aligned} \begin{array}{c} {0.0263}\\ \text{(0.013)}\end{array} \end{aligned}$	$\begin{aligned} \begin{array}{c} {0.0279}\\ \text{(0.035)}\end{array} \end{aligned}$
$\begin{aligned} \begin{array}{c}\text{Log-normal}\\{}\end{array} \end{aligned}$	$\begin{aligned} \begin{array}{c}-0.0055 \\ \text{(0.09)}\end{array} \end{aligned}$	$\begin{aligned} \begin{array}{c}{0.9274}\\ \text{(0.16)}\end{array} \end{aligned}$	$\begin{aligned} \begin{array}{c}{0.0220}\\ \text{(0.011)}\end{array} \end{aligned}$	$\begin{aligned} \begin{array}{c}{0.0279}\\ \text{(0.033)}\end{array} \end{aligned}$	$\begin{aligned} \begin{array}{c} -0.0086 \\ \text{(0.09)}\end{array} \end{aligned}$	$\begin{aligned} \begin{array}{c} {0.9055}\\ \text{(0.15)}\end{array} \end{aligned}$	$\begin{aligned} \begin{array}{c} {0.0259}\\ \text{(0.013)}\end{array} \end{aligned}$	$\begin{aligned} \begin{array}{c} {0.0268}\\ \text{(0.031)}\end{array} \end{aligned}$
$\begin{aligned} \begin{array}{c}\text{Symmetric mixture}\\{}\end{array} \end{aligned}$	$\begin{aligned} \begin{array}{c}{0.0003}\\ \text{(0.09)}\end{array} \end{aligned}$	$\begin{aligned} \begin{array}{c}{1.0046}\\ \text{(0.06)}\end{array} \end{aligned}$	$\begin{aligned} \begin{array}{c}{0.0206}\\ \text{(0.009)}\end{array} \end{aligned}$	$\begin{aligned} \begin{array}{c}{0.0270}\\ \text{(0.031)}\end{array} \end{aligned}$	$\begin{aligned} \begin{array}{c} {0.0005}\\ \text{(0.09)}\end{array} \end{aligned}$	$\begin{aligned} \begin{array}{c} {0.9838}\\ \text{(0.06)}\end{array} \end{aligned}$	$\begin{aligned} \begin{array}{c} {0.0262}\\ \text{(0.012)}\end{array} \end{aligned}$	$\begin{aligned} \begin{array}{c} {0.0264}\\ \text{(0.029)}\end{array} \end{aligned}$
$\begin{aligned} \begin{array}{c}\text{Asymmetric mixture}\\{}\end{array} \end{aligned}$	$\begin{aligned} \begin{array}{c}-0.0008 \\ \text{(0.09)}\end{array} \end{aligned}$	$\begin{aligned} \begin{array}{c}{1.0064}\\ \text{(0.07)}\end{array} \end{aligned}$	$\begin{aligned} \begin{array}{c}{0.0210}\\ \text{(0.010)}\end{array} \end{aligned}$	$\begin{aligned} \begin{array}{c}{0.0245}\\ \text{(0.030)}\end{array} \end{aligned}$	$\begin{aligned} \begin{array}{c} -0.0029 \\ \text{(0.09)}\end{array} \end{aligned}$	$\begin{aligned} \begin{array}{c} {0.9841}\\ \text{(0.07)}\end{array} \end{aligned}$	$\begin{aligned} \begin{array}{c} {0.0269}\\ \text{(0.013)}\end{array} \end{aligned}$	$\begin{aligned} \begin{array}{c} {0.0243}\\ \text{(0.029)}\end{array} \end{aligned}$
$\begin{aligned} \begin{array}{c}\text{Discrete}\\{}\end{array} \end{aligned}$	$\begin{aligned} \begin{array}{c}-0.0004 \\ \text{(0.09)}\end{array} \end{aligned}$	$\begin{aligned} \begin{array}{c}{1.0484}\\ \text{(0.07)}\end{array} \end{aligned}$	$\begin{aligned} \begin{array}{c}{0.0178}\\ \text{(0.010)}\end{array} \end{aligned}$	$\begin{aligned} \begin{array}{c}{0.0332}\\ \text{(0.035)}\end{array} \end{aligned}$	$\begin{aligned} \begin{array}{c}-0.0007 \\ \text{(0.09)}\end{array} \end{aligned}$	$\begin{aligned} \begin{array}{c} {0.9933}\\ \text{(0.07)}\end{array} \end{aligned}$	$\begin{aligned} \begin{array}{c} {0.0280}\\ \text{(0.016)}\end{array} \end{aligned}$	$\begin{aligned} \begin{array}{c} {0.0285}\\ \text{(0.031)}\end{array} \end{aligned}$

**FIGURE 1.**
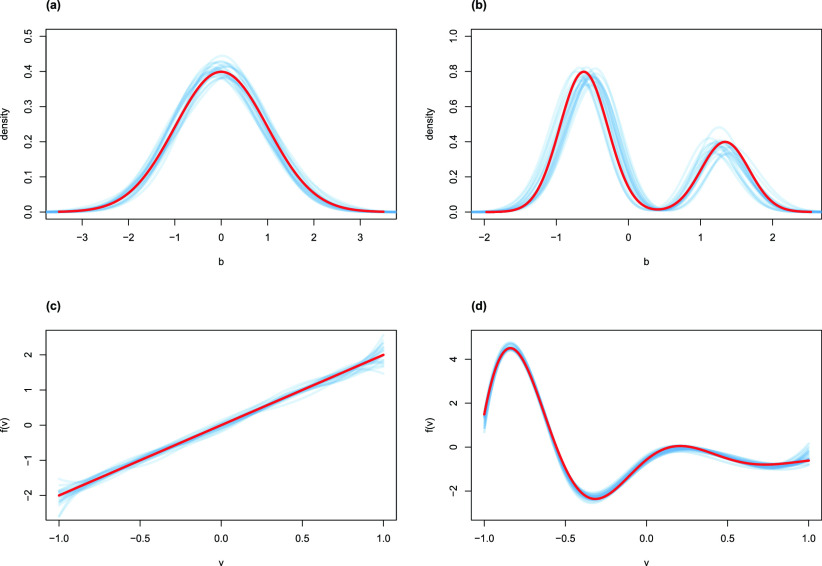
Estimation of hospital effects. For each example, the blue curves is the estimate for replicates, while the true setting with red curve as the benchmark. (a) Standard normal random-effect distribution. (b) Asymmetric random-effect distribution. (c) Linear functional effect $f_{1}$. (d) Nonlinear functional effect $f_{4}$. Each panel includes 20 randomly selected replicated simulations.

As seen in Table 1, the results for both the SNP and normal densities are all close to the true values. However, the latter gives conservative results and suffers from a loss of efficiency, especially when the true $b_{h}$ is drawn from a multimodal or heavy-tailed distribution. This reveals that the normality assumption on $b_{h}$ will prevent the model from capturing all possible heterogeneity across hospitals. In comparison, the SNP approach used in the proposed model (2) estimates the expectation and variance of $b_{h}$ more accurately. At the same time, with respect to various types of the true $b_{h}$, both the ISE for the functional $f(V_{h})$ and the RD for the coefficients of patient effect $\boldsymbol {\beta }$ are quite small in the SNP columns of Table 1. Two components of hospital effects are complementary. Precisely identifying the random effect via the SNP approach can render the estimation of another part unbiased. Figure 1(a, b) depicts the plots of the 20 randomly selected SNP density estimates of $b_{h}$ when setting the true density by the standard normal and asymmetric mixture normal distributions. Using true density (i.e., red curve) as a benchmark, the blue curves show desirable estimates. These results further support the flexibility of the SNP approach.

#### 2) Fixed Effects

Since the SNP random effect exhibits robustness in the proposed hospital compare model (2), the simulations conducted below aim to examine the performance on the estimation of nonparametric $f(V_{h})$. In this scenario, we draw $b_{h}$ from the bimodal distribution $0.7N(-1.5,0.7^{2})+0.3N(2,0.7^{2})$. Four different functions are considered, including polynomial, periodic, and decaying with the attribute $V_{h}$, which follow the $\mathrm {Uniform}(-1,1)$ for $h=1, {\dots },H$. All choices of $f(V_{h})$ are given as follows:

1)
$f_{1}(V_{h}) = 2V_{h}$
2)
$f_{2}(V_{h}) = (V_{h}-0.5)^{2} - 0.5833$
3)
$f_{3}(V_{h}) = V_{h}\cos ^{3}(3V_{h})$
4)
$f_{4}(V_{h}) = e^{-2V_{h}}\sin (6V_{h}) - 0.5748$

We first fit the data using the hospital compare model (2) with the SNP random effect, as defined in (7). This allows us to evaluate the flexibility of functional estimation by B-spline approximation under the different scenarios. The cubic splines of order $d=3$ with equally spaced interior knots $q=3$ are used to approximate all functions $f_{i}(V_{h})$, $i=1, {\dots },4$. Choosing a small $q$ value avoids overfitting. Similar results are obtained when $q$ ranges from 2 to 4; for example, a simple quadratic function $f_{2}$ can be approximated rather well by a quadratic spline basis with $d=2$ and $q=2$. For brevity, we only report in Table 2 the results for $q=3$ with 200 replications. To investigate the impact of hospital fixed-effect misspecification on modeling, by way of comparison, we again fit the data by (2) with the SNP random effect, but now designated $f(V_{h})$ as the linear effect $\alpha V_{h}$. The left side of Table 2 shows the results from the B-spline approximation, while on the right side are those under the linearity assumption.

**TABLE 2:** Simulation Results Under Different Nonparametric Functions, With Standard Deviations in Parentheses

	Nonlinear				Linear			
	$E(b_{h})$	$Var(b_{h})$	ISE	RD ( $\times 10^{-2}$)	$E(b_{h})$	$Var(b_{h})$	ISE	RD ( $\times 10^{-2}$)
Truth	−0.45	3.0625	0	0	−0.45	3.0625	0	0
$\begin{aligned} \begin{array}{c}f_{1} \\{}\end{array} \end{aligned}$	$\begin{aligned} \begin{array}{c}-0.4476 \\ \text{(0.06)}\end{array} \end{aligned}$	$\begin{aligned} \begin{array}{c}{3.0857}\\ \text{(0.14)}\end{array} \end{aligned}$	$\begin{aligned} \begin{array}{c}{0.0087}\\ \text{(0.004)}\end{array} \end{aligned}$	$\begin{aligned} \begin{array}{c}{0.0123}\\ \text{(0.017)}\end{array} \end{aligned}$	$\begin{aligned} \begin{array}{c} -0.4516 \\ \text{(0.09)}\end{array} \end{aligned}$	$\begin{aligned} \begin{array}{c} {3.0785}\\ \text{(0.21)}\end{array} \end{aligned}$	$\begin{aligned} \begin{array}{c} {0.0022}\\ \text{(0.003)}\end{array} \end{aligned}$	$\begin{aligned} \begin{array}{c} {0.0253}\\ \text{(0.029)}\end{array} \end{aligned}$
$\begin{aligned} \begin{array}{c}f_{2} \\{}\end{array} \end{aligned}$	$\begin{aligned} \begin{array}{c}-0.4493 \\ \text{(0.07)}\end{array} \end{aligned}$	$\begin{aligned} \begin{array}{c}{3.0913}\\ \text{(0.14)}\end{array} \end{aligned}$	$\begin{aligned} \begin{array}{c}{0.0074}\\ \text{(0.004)}\end{array} \end{aligned}$	$\begin{aligned} \begin{array}{c}{0.0155}\\ \text{(0.020)}\end{array} \end{aligned}$	$\begin{aligned} \begin{array}{c} -0.4529 \\ \text{(0.09)}\end{array} \end{aligned}$	$\begin{aligned} \begin{array}{c} {3.1493}\\ \text{(0.21)}\end{array} \end{aligned}$	$\begin{aligned} \begin{array}{c} {0.0908}\\ \text{(0.006)}\end{array} \end{aligned}$	$\begin{aligned} \begin{array}{c} {0.0265}\\ \text{(0.036)}\end{array} \end{aligned}$
$\begin{aligned} \begin{array}{c}f_{3} \\{}\end{array} \end{aligned}$	$\begin{aligned} \begin{array}{c}-0.4485 \\ \text{(0.06)}\end{array} \end{aligned}$	$\begin{aligned} \begin{array}{c}{3.0933}\\ \text{(0.1383)}\end{array} \end{aligned}$	$\begin{aligned} \begin{array}{c}{0.0083}\\ \text{(0.004)}\end{array} \end{aligned}$	$\begin{aligned} \begin{array}{c}{0.0129}\\ \text{(0.015)}\end{array} \end{aligned}$	$\begin{aligned} \begin{array}{c} -0.4515 \\ \text{(0.09)}\end{array} \end{aligned}$	$\begin{aligned} \begin{array}{c} {3.1249}\\ \text{(0.20)}\end{array} \end{aligned}$	$\begin{aligned} \begin{array}{c} {0.0510}\\ \text{(0.004)}\end{array} \end{aligned}$	$\begin{aligned} \begin{array}{c} {0.0243}\\ \text{(0.030)}\end{array} \end{aligned}$
$\begin{aligned} \begin{array}{c}f_{4} \\{}\end{array} \end{aligned}$	$\begin{aligned} \begin{array}{c}-0.4461 \\ \text{(0.08)}\end{array} \end{aligned}$	$\begin{aligned} \begin{array}{c}{3.0870}\\ \text{(0.12)}\end{array} \end{aligned}$	$\begin{aligned} \begin{array}{c}{0.0141}\\ \text{(0.006)}\end{array} \end{aligned}$	$\begin{aligned} \begin{array}{c}{0.0142}\\ \text{(0.016)}\end{array} \end{aligned}$	$\begin{aligned} \begin{array}{c} -0.4566 \\ \text{(0.11)}\end{array} \end{aligned}$	$\begin{aligned} \begin{array}{c} {5.4710}\\ \text{(0.41)}\end{array} \end{aligned}$	$\begin{aligned} \begin{array}{c} {2.4043}\\ \text{(0.120)}\end{array} \end{aligned}$	$\begin{aligned} \begin{array}{c} {0.0278}\\ \text{(0.034)}\end{array} \end{aligned}$

When the true hospital fixed effect is linear $f_{1}$, fitting by linear specification $\alpha V_{h}$ is naturally in efficiency. In using the B-spline method to estimate the functional effect, the left side of Table 2 shows that the estimation of $\boldsymbol {\beta }$ and $f(V_{h})$ is unbiased in all four cases, with the estimates for the expectation and variance of $b_{h}$ close to their true values. However, under an inappropriate and simple hospital fixed-effect specification $\alpha V_{h}$, the results of examined performance metrics are compromised. In particular, as seen in the right side of Table 2, when $f_{4}$ has a significant nonlinear pattern with respect to the attribute $V_{h}$, the hospital effects produce erroneous estimates. This reveals that the linearity assumption for the hospital fixed effect makes it difficult to detect the complex relationship between response and explanatory variables, which inevitably affects the estimation of other components in the mean structure. To visualize the B-spline approximation effects, the 20 randomly selected B-spline estimates $\hat {f}_{1}(V_{h})$ and $\hat {f}_{4}(V_{h})$, marked with blue color smoothed curves, are shown in Figure 1(c, d). They precisely depict the trend of the true curve (the red curve), which provide further evidence of the flexibility by the nonlinear modeling approach. In particular, the linear trend in $f_{1}$ can be accurately captured as depicted in Figure 1(c).

### B. Utility of ZIB Hospital Compare Models

The simulations in Section V-A confirm the robustness of the proposed hospital compare model (2) for estimating the distribution of the random effect and the functional form of the fixed effect. Yet, when there are excessive zeros in the patient responses, we need to employ the ZIB hospital compare models (9) –(10) proposed in Section III-B to properly fit such unbalanced data with excessive zeros, in order to demonstrate the generality of our proposed modeling framework. In this simulation, we assume the data are generated from the following two-status model. One models the probability of the status for being at risk of mortality with $S_{hj}=1$ as

\begin{equation*} \log \left ({\frac {P\left ({S_{hj}=1 \mid \boldsymbol {X}_{hj}}\right)}{1-P\left ({S_{hj}=1 \mid \boldsymbol {X}_{hj}}\right)}}\right)= {X}^{\top }_{hj1}\theta \end{equation*}

for $j=1, {\dots }, n_{h}$ and $h=1, {\dots }, H$. Here, we set $H=500$ and $n_{h}=100$. The value of $\theta $ controls the average proportion of positive events (APE) with $Y_{hj}=1$. The other models the probability of the status of mortality with $Y_{hj}=1$ as follows:

\begin{align*} \begin{cases} \displaystyle \log \left ({\frac {P\left ({Y_{hj}=1 \mid \boldsymbol {X}_{hj}, S_{hj}}\right)}{1-P\left ({Y_{hj}=1 \mid \boldsymbol {X}_{hj}, S_{hj}}\right)}}\right)\!=\! b_{h} \!+\! f(V_{h}) \!+\! \boldsymbol {X}_{hj}^{\top } \boldsymbol {\beta }, \\[0.4pc] \displaystyle \qquad \quad { \text {if }} S_{hj}=1, \\[0.2pc] \displaystyle P\left ({Y_{hj}=1 \mid \boldsymbol {X}_{hj}, S_{hj}}\right)=0, \\[0.4pc] \displaystyle \qquad \quad { \text {if }} S_{hj}=0, \end{cases} \end{align*}

where $\boldsymbol {\beta } = (\beta _{1},\beta _{2},\beta _{3})^{\top }=(3, 0.5, -5)^{\top }$, $\boldsymbol {X}_{hj}^{\top }=(X_{hj1},X_{hj2},X_{hj3})$ are generated from $N(0, \boldsymbol {I}_{3})$; the functional fixed effect $f(V_{h})$ is specified as $e^{-2V_{h}}\sin (6V_{h}) - 0.5748$ with $V_{h}\sim \mathrm {Uniform}(-1,1)$; and $b_{h}$ is generated by standardizing the bimodal distribution $0.7N(-1.5,0.7^{2})+0.3N(2,0.7^{2})$.

To generate the data in which responses have excessive zeros, for each individual patient, we set $\theta =2$ and $\theta =-2$ with $X_{hj1}$ generated from $N(1, 1.5^{2})$ to control the APE so it is 32% and 11%, respectively. As described in Section III-B, for each realization $(y_{hj}, \boldsymbol {x}_{hj}, v_{h})$, $s_{hj}$, $s_{hj}$ is considered as unknown if $y_{hj}=0$. The simulation data are divided into training and test data sets of equal size within each cluster for further evaluation. We use the proposed ZIB hospital compare additive models (9) –(10), denoted by “ZI-HCAM”, and the proposed hospital compare additive models without any zero-inflated structure (2), denoted by “HCAM”, to fit the training data. Regarding the ZI-HCAM, all of its covariates $\boldsymbol {x}_{hj}$ are used to fit the two statuses given by (9) and (10). The area under the receiver operating characteristic curve (AUC) is calculated after fitting the test data set. To evaluate the fitting performance of the above two models, five metrics are used: the estimated mean and variance of $b_{h}$, ISE, RD, and AUC. We record the results of 200 replications for each model and report the average results of those metrics in Table 3.

**TABLE 3:** Results of Different Configurations When the Data Have Excessive Zeros. Standard Deviations are Shown in Parentheses

Model	$E(b_{h})$	$Var(b_{h})$	ISE	RD ( $\times 10^{-2}$)	AUC
Truth	0	1	0	0	1
APE =32%				
$\begin{aligned} \begin{array}{c}\text{ZI-HCAM}\\{}\end{array} \end{aligned}$	$\begin{aligned} \begin{array}{c}{0.0833}\\ \text{(0.12)}\end{array} \end{aligned}$	$\begin{aligned} \begin{array}{c}{1.0168}\\ \text{(0.21)}\end{array} \end{aligned}$	$\begin{aligned} \begin{array}{c}{0.0383}\\ \text{(0.023)}\end{array} \end{aligned}$	$\begin{aligned} \begin{array}{c}{0.2251}\\ \text{(0.267)}\end{array} \end{aligned}$	$\begin{aligned} \begin{array}{c}{0.9336}\\ \text{(0.003)}\end{array} \end{aligned}$
$\begin{aligned} \begin{array}{c}\text{HCAM}\\{}\end{array} \end{aligned}$	$\begin{aligned} \begin{array}{c}-1.6446 \\ \text{(0.05)}\end{array} \end{aligned}$	$\begin{aligned} \begin{array}{c}{0.0858}\\ \text{(0.02)}\end{array} \end{aligned}$	$\begin{aligned} \begin{array}{c}{1.7905}\\ \text{(0.142)}\end{array} \end{aligned}$	$\begin{aligned} \begin{array}{c}{38.3643}\\ \text{(0.721)}\end{array} \end{aligned}$	$\begin{aligned} \begin{array}{c}{0.9015}\\ \text{(0.003)}\end{array} \end{aligned}$
APE = 11%				
$\begin{aligned} \begin{array}{c}\text{ZI-HCAM}\\{}\end{array} \end{aligned}$	$\begin{aligned} \begin{array}{c}-0.0129 \\ \text{(0.12)}\end{array} \end{aligned}$	$\begin{aligned} \begin{array}{c}{0.9857}\\ \text{(0.24)}\end{array} \end{aligned}$	$\begin{aligned} \begin{array}{c}{0.0377}\\ \text{(0.019)}\end{array} \end{aligned}$	$\begin{aligned} \begin{array}{c}{0.2188}\\ \text{(0.281)}\end{array} \end{aligned}$	$\begin{aligned} \begin{array}{c}{0.9114}\\ \text{(0.003)}\end{array} \end{aligned}$
$\begin{aligned} \begin{array}{c}\text{HCAM}\\{}\end{array} \end{aligned}$	$\begin{aligned} \begin{array}{c}-2.4121 \\ \text{(0.04)}\end{array} \end{aligned}$	$\begin{aligned} \begin{array}{c}{0.0533}\\ \text{(0.03)}\end{array} \end{aligned}$	$\begin{aligned} \begin{array}{c}{2.2822}\\ \text{(0.139)}\end{array} \end{aligned}$	$\begin{aligned} \begin{array}{c}{88.1523}\\ \text{(0.773)}\end{array} \end{aligned}$	$\begin{aligned} \begin{array}{c}{0.8455}\\ \text{(0.003)}\end{array} \end{aligned}$

From Table 3, it is evident that $E(b_{h})$ and $Var(b_{h})$ are reasonably estimated by the ZI-HCAM since they lie near their true values. Further, the metrics ISE and RD are also close to zero, which implies the estimates of $f(V_{h})$ and $\boldsymbol {\beta }$ are very close to their true values also. Given its higher AUC, the ZI-HCAM is more robust for prediction. By way of comparison, the HCAM performs worse, especially when the APE drops. This indicates that the proposed ZIB hospital compare model (9) –(10) is superior for analyzing the zero-inflated data.

## VI. Experiments on the ICU Mortality Data In U.S. Hospitals

### A. Data Description

We acquire the data from Women in Data Science (WiDS) Datathon 2020 [26], an open-access data set—based upon the Massachusetts Institute of Technology’s Global Open Source Severity of Illness Score (GOSSIS) database—that contains groups of features, including identifiers, patient demographics, acute physiology and chronic health evaluation (APACHE) scores, laboratory results, and various vital signs. The minimum and maximum values from clinical monitoring for the first hour and first 24 hours after admission to the ICU are recorded. We now shift our focus to comparing hospital performance on the basis of their medical and surgical ICUs to eliminate the heterogeneities in different types of units. In fact, the patients admitted to two types of ICUs account for nearly 70% of the recorded total observations. Following recent work on measuring feature importance for predicting mortality [27], [28], we select the related variables and summarize their descriptions in Table 4. All these variables are recognized as important predictors of mortality in patients.

**TABLE 4:** Description of the Predictor Variables Used in This Study

Variable	Description
$death$	Whether the death event occurred. $y_{hj}=1$ if $j$-th patient in $h$-th hospital died in the ICU.
$age$	The age of the patient in years.
$BMI$	Body mass index of the patient.
$elesur$	Whether the patient was admitted to the hospital for an elective surgical operation.
$stay$	Whether the patient was admitted to the icu as readmit or transfer type.
$days$	The length of stay of the patient between hospital admission and unit admission.
$GCS$	The Glasgow Coma Scale (eyes, motor and verbal) measured during the first 24 hours which results in the highest APACHE III score.
$heartrate$	The patient’s average APACHE III score of extremums of heart rate during the first 24 hours of the unit stay.
$resprate$	The patient’s average APACHE III score of extremums of respiratory rate during the first 24 hours of the unit stay.
$temp$	The patient’s core temperature score during the first 24 hours of their unit stay. Calculated by the average of $(\text {extremum}-36.5)^{2}$.
$bun$	The average of extremums of blood urea nitrogen concentration of the patient in their serum or plasma during the first 24 hours of the unit stay.
$arf$	Whether the patient had acute renal failure during the first 24 hours of their unit stay.
$intubated$	Whether the patient was intubated at the time of the highest scoring arterial blood gas used in the oxygenation score.
$ventilated$	Whether the patient was invasively ventilated at the time of the highest scoring arterial blood gas using the oxygenation scoring algorithm, including any mode of positive pressure ventilation delivered through a circuit attached to an endotracheal tube or tracheostomy.
$comorbidity$	Whether the patient has been diagnosed with some associated comorbidities, such as cirrhosis, aids, diabetes, and so on.
$volume$	The number of the observed admissions averaged by ICU at hospital $h$.

Records during the first 24 hours of the patients’ ICU stays are revised by the minimal and maximal records of daily and hourly clinical monitoring. Missing values and those cases from 16 hospitals with no patient deaths (cases of $y_{hj}=1$) during the observation period are excluded in our study. The pre-processed data set records the instances for 47852 patients treated in 124 hospitals. Following the notations in Section II, each case contains an indicator of whether a patient died ( $y_{hj}=1$) after being admitted to the ICU, patient-specific attributes (such as age, body mass index or BMI, APACHE scores, etc.), and a hospital-specific attribute (i.e., hospital volume). Within this data set, the total number of admitted patients per hospital $n_{h}$ varies greatly, ranging from 3 to 1153, with a median value of 167. The observed mortality rate, $OR_{h} = n_{h}^{-1}\sum _{j} y_{hj}$, is distributed between 0.88% to 28.30%, and its overall average is 8.33%.

**FIGURE 2.**
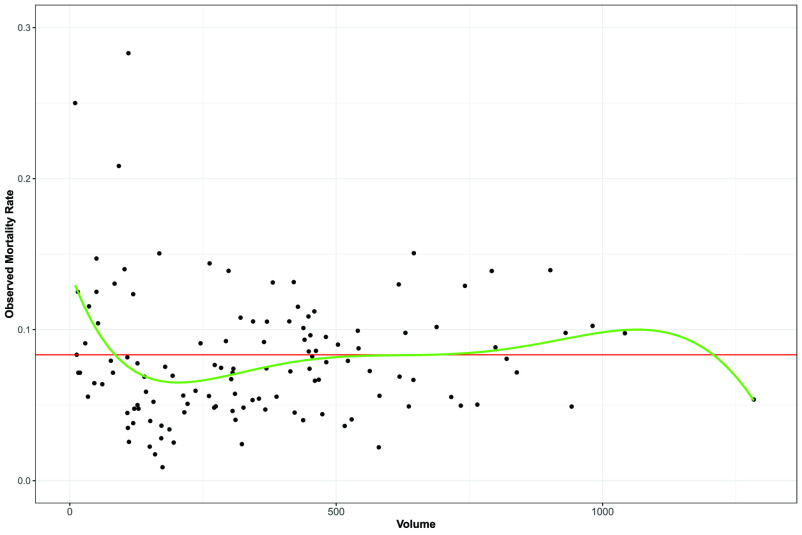
Observed mortality rates by volume (the number of admitted patients). The red horizontal line shows the overall observed rate of 0.0833. The green smoothing curve indicates the rate averaged by volume.

Figure 2 plots the observed mortality rate by $volume$, the hospital attribute of interest, for all death events occurring in the ICU at hospital $h$. The overall average rate (red horizontal line) only represents the average risk, while the green smoothed curve showing a rapid downward trend at smaller volumes and a gentle upward trend at larger volumes. The mortality rate in some low-volume hospitals is considerably higher than the overall average, which could imply their potential treatment risk. When the $volume$ is around 200, we observe an aggregation of points representing the hospitals with relatively low mortality rates. We may thus speculate that the high-volume hospitals deliver more reliable and effective treatment and care, which results in an average mortality rate. On the other hand, the healthier patients’ physical conditions are treated by a hospital, the fewer deaths may occur.

**FIGURE 3.**
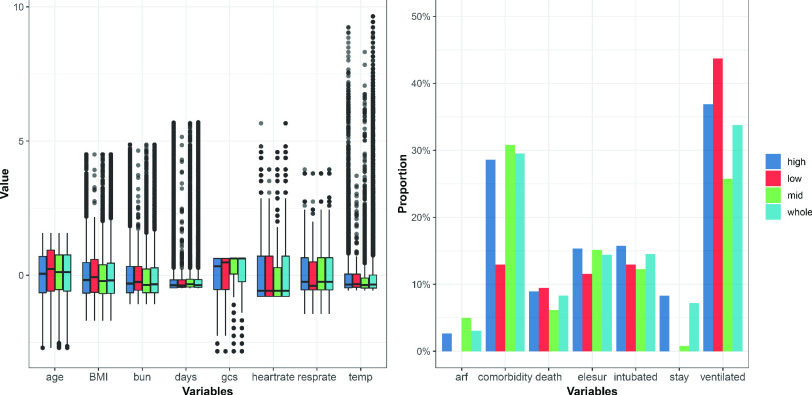
Box plots and bar plots for the various patient characteristics.

In Figure 3 are box plots and bar plots of the patient characteristics vis-à-vis different volume levels; to the left are continuous attributes and to the right the binary attributes. To compare with the whole, we screened out three subsets of data in which the $volume$ is less than 65 (10% quantile), between 300 and 360 (45% to 55% quantile), and greater than 728 (90% quantile), respectively. As evinced by Figure 3, there are substantial differences in the attributes’ distribution across those three levels. For instance, in the low-volume hospitals, the patients have higher values that are recorded for continuous variables and have a higher proportion for the variable $ventilated$.

To provide a reasonable evaluation of death events in these hospitals and to understand the reasons behind variation in their health outcomes, we suggest adjusting the mortality risk by improving the hospital compare models. However, the classical GLMMs are not robust for this task given the complexity of large-scale hospital compare data. Instead, we recommend applying the proposed hospital compare models (2) and (9) –(10) to reveal the information behind the data. Baseline demographics, clinical variables, and hospital attributes are compared between the patients with and without deaths using our proposed models.

### B. Evaluation of the Proposed Hospital Compare Models

In this subsection, we investigate the performance of two types of proposed hospital compare models, namely (2) and (9) –(10) incorporating the flexible SNP random effect mentioned in Section III, these respectively abbreviated as “HCAM” (hospital compare additive model, with additive hospital fixed effects) and “ZI-HCAM” (zero-inflated hospital compare additive model, with a zero-inflated structure). To model the varied effects across groups, the hospital attribute chosen in $volume$. The other 14 important patient characteristics are entered as predictors to explain the response variable, $death$. Regarding the ZI-HCAM, risk-free mortality is a manifestation of individual immunity modeled by eight variables: $age$, $BMI$, $GCS$, $heartrate$, $resprate$, $temp$, $bun$, and $comorbidity$ (Table 4). To further compare the performance of different models in terms of inference and prediction, the GLMM (1) with linear hospital fixed effect $\alpha V_{h}$ is applied as well. The observations are randomly split into a training data set (2/3) and a test data set (1/3) within each hospital.

Unless no differences among hospitals exist, an SNP random effect can measure this heterogeneity more flexibly than a standard Gaussian random effect. We use the following performance evaluation metrics to compare above three models: AUC with 95% confidence intervals (CIs), accuracy, F_1_-score, and positive predictive value (PPV). In addition to the AUC, the observed overall average rate of 0.0833 is determined as a unified threshold to calculate the above measures.

The results for the above diagnostic measures are presented in Table 5, while the estimated expectations and variances of $b_{h}$ (with the standard error in parentheses) are also displayed in the last two rows. As the benchmark, the GLMM’s AUC and corresponding 95% CI was 0.840 (0.830, 0.851) in the test data set. Applying the HCAM, we find that its performance is nearly identical to the GLMM, with an AUC of 0.839 (0.829, 0.850). In stark contrast, using the ZI-HCAM gives a significant improvement in performance, with an AUC of 0.851 (0.841, 0.862). The prediction performance of the HCAM is comparable to the GLMM in terms of the AUC, but slightly higher in terms of accuracy, F_1_-score, and PPV. This indicates the HCAM is a more refined model than the GLMM, that is, when not assuming a linear relationship for the hospital fixed effect and a Gaussian type of random effect. Since the proportion of deaths in such medical records is exceedingly low, both the GLMM and HCAM lead to inconsistent parameter estimation and uncertainty in classification ability; therefore, the ZI-HCAM outperforms them.

**TABLE 5:** Comparison and Summary Results For the Prediction Performance of Random-Effects Models

Model	GLMM	HCAM	ZI-HCAM
AUC (CI)	0.840	0.839	0.851
	(0.830, 0.851)	(0.829, 0.850)	(0.841, 0.862)
Accuracy(%)	76.03	76.67	78.62
F_1_-score(%)	34.42	34.83	35.95
PPV(%)	22.39	22.69	23.95
$E(b_{h})$	−3.4002	−3.4222	−2.7074
	(0.0950)	(0.0558)	(0.0606)
$Var(b_{h})$	0.1002	0.0858	0.1059
	(0.0250)	(0.0234)	(0.0259)

**FIGURE 4.**
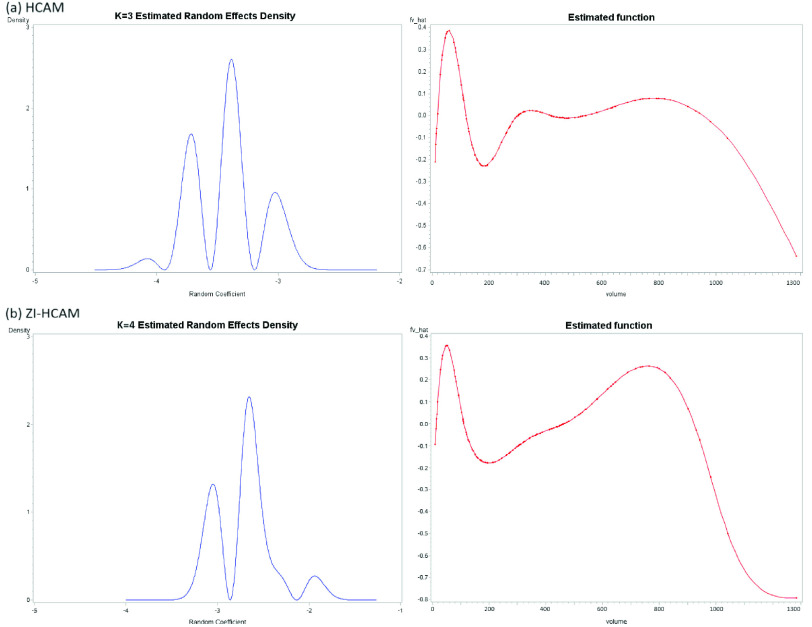
The estimated hospital random effect $b_{h}$ and the fixed effect $f(V_{h})$.

**FIGURE 5.**
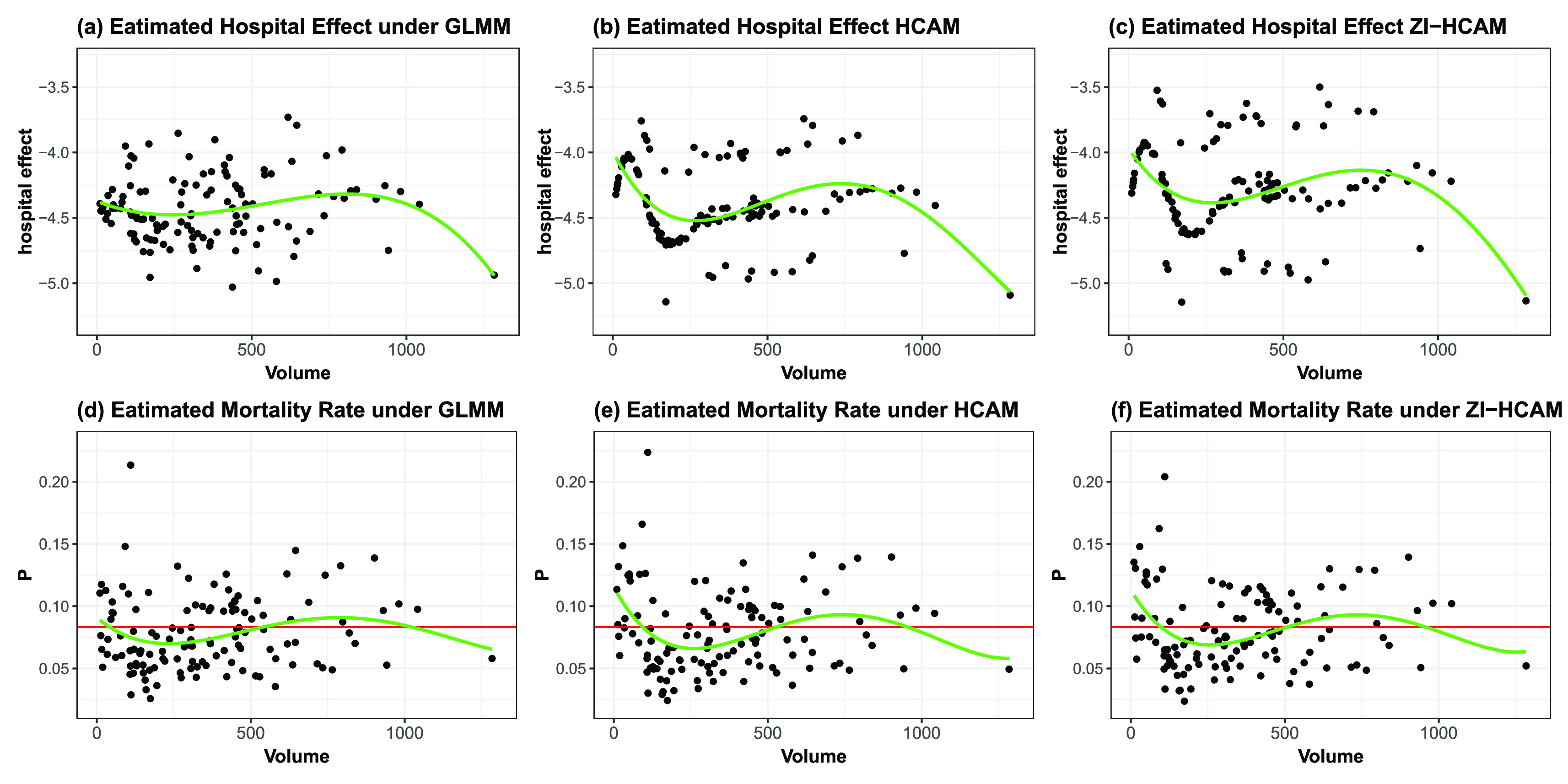
Plots of the estimated hospital effect (a–c) and the estimated average expected mortality rate (d–f) versus volume (the number of admitted patients). Green smoothing curves indicate the estimated value averaged by volume. Red horizontal lines in (d–f) show the overall observed rate of 0.0833.

Model assessment entails more than just forecasting performance, however. The newly proposed models (2) and (9) –(10) could fully capture information in the data by relaxing assumptions about the random-effect distribution and the structure of the hospital fixed effect. Figure 5 shows the estimated density of $b_{h}$ and the relationship between hospital attribute $volume$ and response $death$ for the three models. As presented in Figure 4, the parameter $K$ governs the adaptability of an SNP $b_{h}$ and provides the optimal selection with a multimodal shape for both the HCAM and ZI-HCAM. The estimated function $\hat {f}$ (red curve) has a nonlinear relationship with the attribute $volume$, whose trend is similar to the green curve in Figure 2. The estimated hospital fixed effect $\hat {f}$ is exceptionally high in hospitals with small volumes, tends to be flat in medium-sized hospitals, and undergoes rapidly decline in larger hospitals. When the volume increases from about 200 to 800, the corresponding $\hat {f}$ gradually rises as well. This suggests the potential quality of healthcare in large-volume hospitals may not be as good as that of hospitals having medium volumes.

### C. Inference on Mortality Rates In Hospital Compare

Precise evaluation of the hospital mortality rates is the target of developing a hospital compare model. Using the acquired data set, we can arrive at a preliminary inference on the hospital mortality rates. Let $P_{h}$ be the average expected mortality rate for hospital $h$ with $P_{h} = n_{h}^{-1} \sum _{j=1}^{n_{h}} p_{h}(\boldsymbol {X}_{hj})$, in which $\mathcal {H} = h$ and $\boldsymbol {\mathcal {X}}= \boldsymbol {X}_{hj}$ in (13). Similar to (11), we define the individual expected mortality rate using ZI-HCAM as $p_{h}(\boldsymbol {X}_{hj}, \boldsymbol {T}_{hj})=\pi _{h}(\boldsymbol {X}_{hj})\rho (\boldsymbol {T}_{hj})$, where $\pi _{h}(\boldsymbol {X}_{hj}) = \text {logit}^{-1} (b_{h} + f(V_{h}) + \boldsymbol {X}_{hj}^{\top } \boldsymbol {\beta })$ and $\rho (\boldsymbol {T}_{hj})=\text {logit}^{-1} (\boldsymbol {T}_{hj}^{\top } \boldsymbol {\theta })$. Figure 5 plots the estimated hospital effect and $P_{h}$ for fitted the GLMM, HCAM, and ZI-HCAM.

The green smoothed curves in Figure 5(a-c) are trends for the average of estimated hospital effects across $volume$, revealing the variability in estimated mortality rates. Compared with Figure 5(a), significant variation appears in the scatter for estimated hospital effects in Figure 5(b) and 5(c) distributed on both sides of the green smoothing curve. This is because the hospital fixed effect specifies the macro trend with changes to $volume$, and the estimated multimodal random effect divides this tendency into three groups to identify their corresponding micro trend. Referring to the estimated coefficient $\alpha =0.0514$ (standard error $= 0.1601$) of the hospital attribute $volume$ under the GLMM, the higher mortality rates at the lower-volume hospitals are more due to a riskier patient case-mix rather than variation in the hospital effect per se. Yet, Figure 5(e) and 5(f) depict that the estimated mortality rates in many smaller-volume hospitals are larger than 0.12, which points to hazards inherent in these hospitals. Estimates of the ZI-HCAM are adjusted from the HCAM after considering the risk-free patients. The average predicted mortality rate among the hospitals increases from 0.0793 to 0.0808, while for the GLMM it is 0.0779.

**FIGURE 6.**
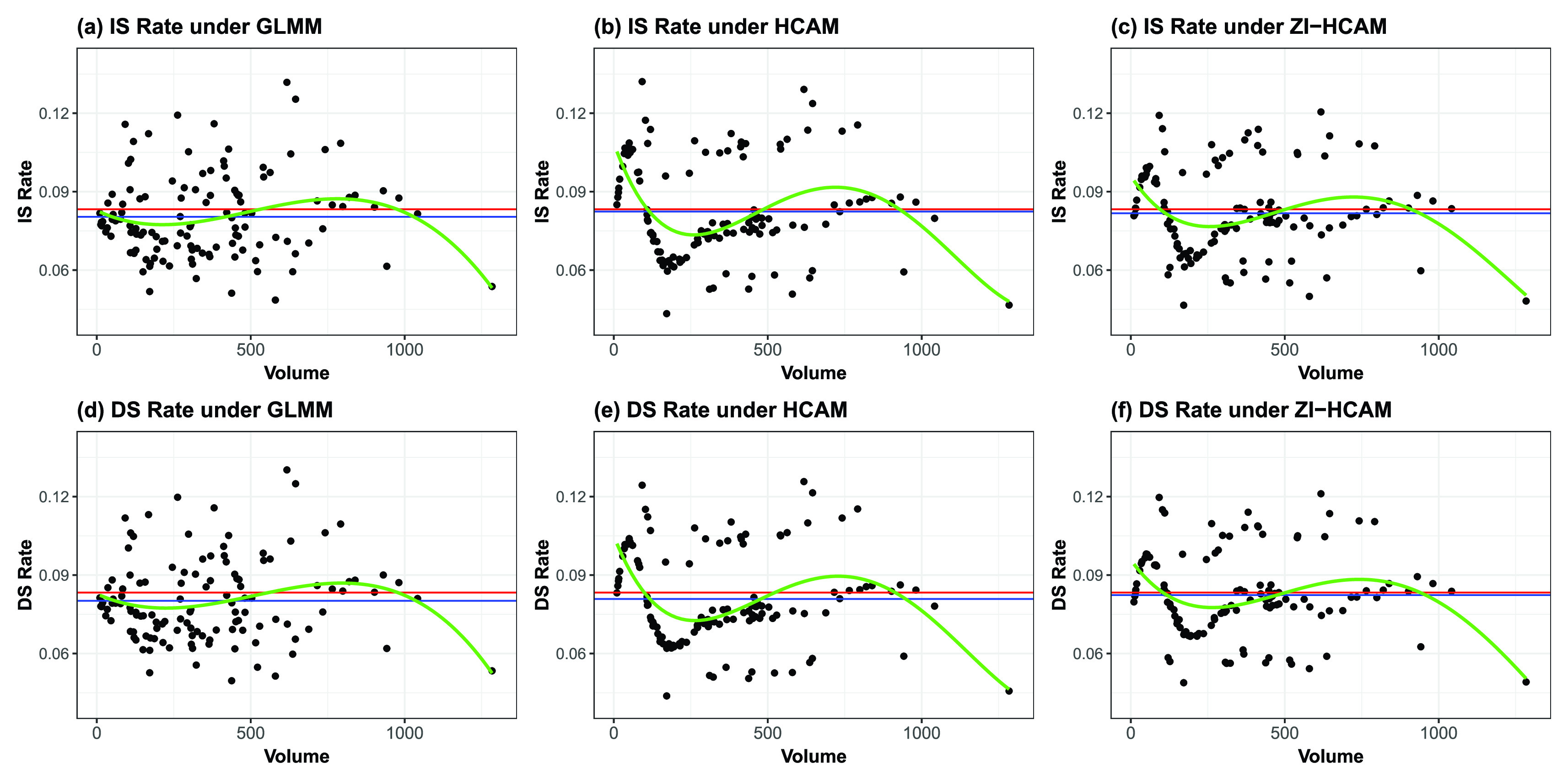
Estimated standardized rates, $\hat {P}_{h}^{IS}$ or $\hat {P}_{h}^{DS}$,by volume. Red horizontal lines show the overall observed rate of 0.0833. Blue horizontal lines indicate the simple average of the standardized rates. Green smoothing curves indicate the estimated the indirectly standardized (IS) mortality rate and directly standardized (DS) mortality rate averaged by volume (the number of admitted patients).

The primary purpose of carrying out a hospital compare analysis is to estimate and standardize the mortality rate by adjusting patient case-mix variation. Two mortality rate standardization methods reviewed in Section IV are implemented here to comprehensively evaluate expected risks and to rank hospitals accordingly. Treating the standardized rate from the GLMM as the benchmark, the indirectly standardized (IS) mortality rate and directly standardized (DS) mortality rate are each determined for the HCAM, as well as for the ZI-HCAM. Corresponding to (11) and (13), $\hat {P}_{h}^{IS}$ and $\hat {P}_{h}^{DS}$ for the ZI-HCAM is calculated by $p_{\mathcal {H}}(\boldsymbol {X}_{hj}, \boldsymbol {T}_{hj}) = \pi _{\mathcal {H}}(\boldsymbol {X}_{hj}) \rho (\boldsymbol {T}_{hj})$, as if the $hj$-th patient had been treated at hospital $\mathcal {H}$, where $\pi _{\mathcal {H}}(\boldsymbol {X}_{hj}) = \text {logit}^{-1} (b_{\mathcal {H}} + f(V_{\mathcal {H}}) + \boldsymbol {X}^{\top }_{hj} \boldsymbol {\beta })$, $\rho (\boldsymbol {T}_{hj})=\text {logit}^{-1} (\boldsymbol {T}^{\top }_{hj} \boldsymbol {\theta })$. The estimated $\hat {P}_{h}^{IS}$ and $\hat {P}_{h}^{DS}$ for each of the three models are plotted in Figure 6. Here, we use the weighted expected mortality $E_{h}$ as defined in (16), to measure $\hat {P}_{h}^{IS}$. The weights take $w_{h} = n_{h} / N$, where $N=\sum _{h}n_{h}$.

Intuitively, these $\hat {P}_{h}^{IS}\text{s}$ shrink the $\hat {P}_{h}\text{s}$ to resemble their corresponding hospital effects in Figure 5. At thesame time, $\hat {P}_{h}^{IS}$ and $\hat {P}_{h}^{DS}$ are characterized by a similar trend in Figure 6. Specifically, the risk-adjusted mortality rate is generally higher in low-volume hospitals for the proposed HCAM and ZI-HCAM, irrespective of the indirect and direct standardization of mortality rates used. Low-volume hospitals expose substantial risk. This risk presents a clear downward sloping trend as the $volume$ increased to about 200. It differs from the GLMM whose indirect and direct standardization of mortality rates instead show a gentle trend. Another phenomenon is that some medium-volume hospitals harbor the same adjusted risk as low-volume hospitals. The average of $\hat {P}_{h}^{IS}$ and $\hat {P}_{h}^{DS}$ (the blue horizontals) rises above the overall average mortality rate (the red horizontals). This indicates that the observed rate for all patients essentially understates the baseline risk.

Certainly, patients tend to seek medical care in larger-volume hospitals due to the latter’s perceived lower risk of death. Referring to George et al. [11], while the indirectly standardized mortality rates does have shortcomings when trying to eliminate the effect of patient case-mix variation, it is still a worthwhile alternative since the patient’s selection is more realistic. The proposed weighted indirectly standardized mortality rates adjust the probability of a patient’s decision to choose a given hospital by weighting $E_{h}$. By contrast, direct standardization of mortality rates uses a fixed patient population to fairly compare hospitals. The estimated weighted indirectly standardized mortality rates have strong linear correlations with the estimated directly standardized mortality rates, with correlation coefficients of 0.9971, 0.9968, and 0.9930 under the above GLMM, HCAM, and ZI-HCAM, respectively. Therefore, we only include the directly standardized mortality rates $\hat {P}_{h}^{DS}$ for these 124 hospitals in the below comparison and their ranking.

**FIGURE 7.**
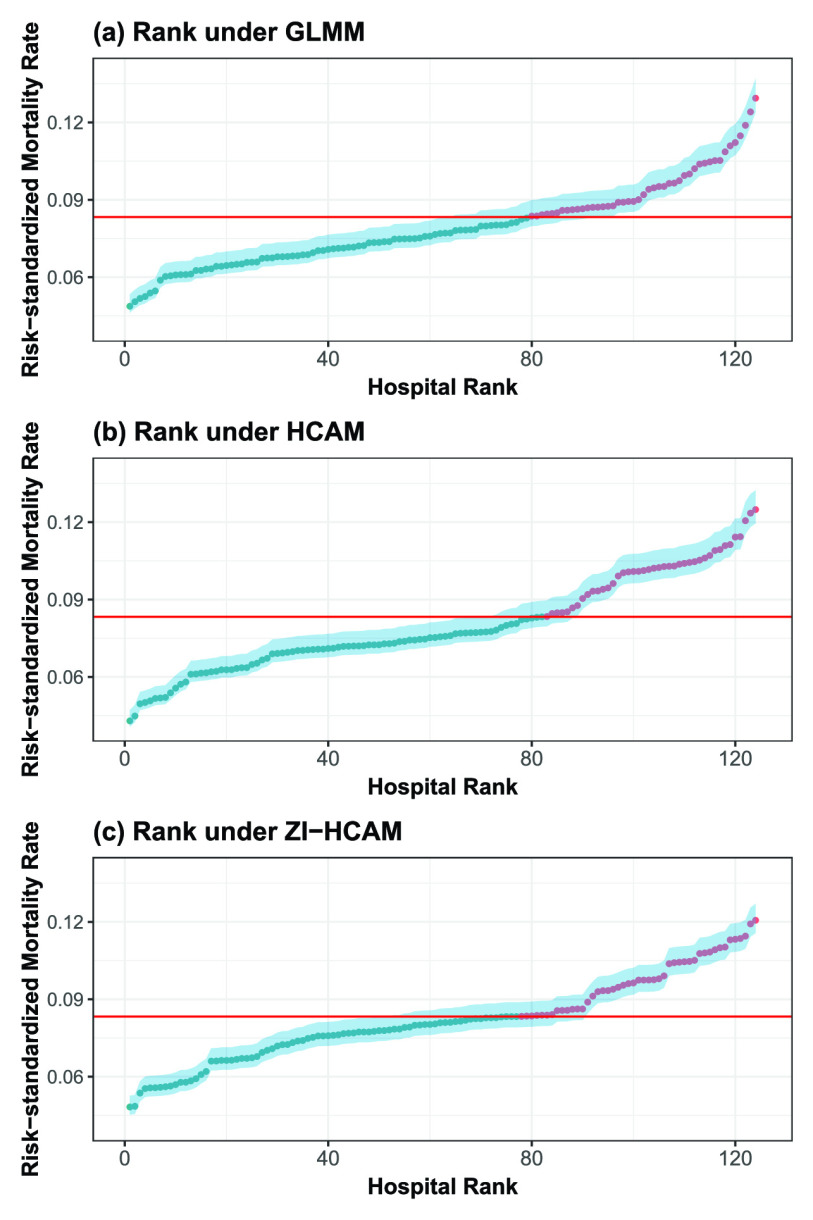
Risk-standardized mortality rates $\hat {P}_{h}^{DS}$ forthree models. Red horizontal lines represent the overall observed rate observed, i.e. 0.0833. Dark blue dots represent those hospitals with a standardized mortality rate below the overall observed rate; conversely, red dots represent those above the overall observed rate. The shaded area indicates the 95% bootstrap confidence intervals for the directly standardized mortality rates estimates.

By applying the bootstrap method—using 2000 bootstrapped samples, each resampling 2000 patient cases without replacement—we calculate the confidence interval for the directly standardized mortality rates $\hat {P}_{h}^{DS}$. As seen in Figure 7, $\hat {P}_{h}^{DS}$ varies considerably across the hospitals, ranging from 0.0487 to 0.1294 for the GLMM; from 0.0429 to 0.1249 for the HCAM; and from 0.0482 to 0.1206 for the ZI-HCAM. The directly standardized mortality rates in more than a third of the hospitals is greater than the overall average of 0.0833. Unlike the GLMM, the directly standardized mortality rates from our proposed HCAM and ZI-HCAM present higher risks for those hospitals ranked near the bottom. This may imply that these hospitals’ standard of medical service and care is inferior; hence, the patients admitted to hospitals with low rankings are more vulnerable to death. Compared with the GLMM, a majority of hospitals change in rank: 121 for the HCAM (or 97.6%) and 153 for the ZI-HCAM (or 93.5%), respectively. The corresponding median of absolute rank changes is 15 and 13, respectively.

**FIGURE 8.**
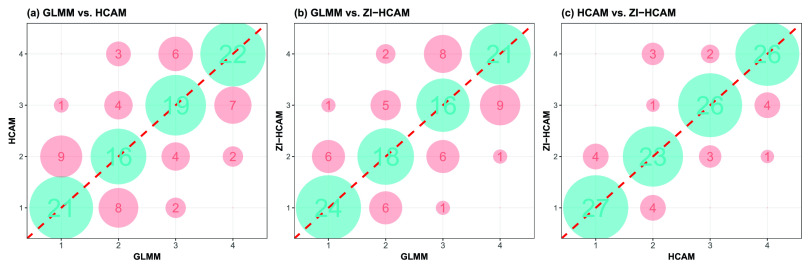
Comparison of hospital rankings in quartiles between the GLMM, HCAM, and ZI-HCAM. Each axis represents the risk-adjustment model used in this study. The number in each circle indicates the number of hospitals that fall into the quartiles shown on each axis, spanning the best-performing quartile “1” to the worst-performing quartile “4.”

We use quartiles to distinguish the ranking outcomes of the three models. As Figure 8 shows, utilizing the ranks from the GLMM as a benchmark, 46 and 45 hospitals undergo revision by the HCAM and ZI-HCAM, corresponding to 37.10% and 36.29% of all hospitals, respectively. Among these changes, 23 and 22 hospitals are reclassified into the lower-performing quartile according to the HCAM and ZI-HCAM, respectively. Notably, the ranking of four hospitals based on HCAM is adjusted downward by two quartiles. The ranks of the proposed HCAM and ZI-HCAM give similar results, both in Figure 7(b, c) and Figure 8(b, c). As a model with better predictability, the ZI-HCAM revises the ranking given by the HCAM.

## VII. Discussion

The classical GLMMs have difficulty capturing nonnormal random effects, nonlinearity in conditional mean structures, and excessive zeros. Inspired by the zero-inflated structure, in this article, we propose a novel framework to model and analyze hospital compare data, which combines the flexibility of functional terms for the hospital fixed effect and the SNP random hospital effects. Our proposed models (2) and (9) –(10) were evaluated against the classical GLMMs with respect to predictability and interpretability. In general, the flexible SNP random effect reduces errors that arise from violation of normality assumptions in random effects. The nonparametric mean structure, as opposed to a simple linear representation, reliably captures the information closest to the underlying truth.

The value of our work lies in that it is the first time that the semi-nonparametric approach and zero-inflated structure are combined and utilized to build and improve the hospital compare models. The solutions of the proposed models are founded on the $\mathtt {SAS}$ platform, with the help of semi-parametric and semi-nonparametric methods to specify the fixed and random effects, respectively. Hence, we provide the corresponding $\mathtt {SAS}$ programs for their convenient implementation and application.

While we have built a modeling framework containing linear additive terms, there remains room to strengthen the proposed models. Usually, a comprehensive evaluation of one hospital depends on measuring the effects from multiple hospital-associated attributes, such as the number of beds, the number of nursing staff, etc. Consequently, several additive forms of hospital fixed effect $\sum ^{p}_{i=1} f_{i}(V_{i,h})$ could be available in model (2), in which $p$ denotes the dimensions for the hospital attributes of interest. In addition, the hospital fixed effects, denoted by $\boldsymbol {V}_{h}=(V_{1,h}, {\dots }, V_{p,h})^{\top }$, with varying coefficient terms [29] or even single index terms [30], could also be considered in model (2). We can still use the B-spline method to approximate the above nonparameteric terms. This will expand the applicability and optionality of the framework.
